# Mycotic infections – mucormycosis and oral candidiasis associated with Covid-19: a significant and challenging association

**DOI:** 10.1080/20002297.2021.1967699

**Published:** 2021-08-26

**Authors:** Manjusha Nambiar, Sudhir Rama Varma, Mohamed Jaber, S. V. Sreelatha, Biju Thomas, Arathi S. Nair

**Affiliations:** a Department of Periodontics, College of Dentistry, Sri Rajiv Gandhi, College of Dental Sciences and Hospital, Benguluru, India; b Department of Clinical Sciences, College of Dentistry, Ajman University, Ajman, UAE; c Centre of Medical and Bio-allied Health Sciences Research, Ajman University, Ajman, UAE; d Department of Oral and Maxillofacial Surgery, College of Dentistry, Ajman University, Ajman, UAE; e Department of Oral Pathology, College of Dentistry, Ab Shetty Institute of Dental Sciences- (Nitte to Be Deemed University), Manguluru, India; f Department of Periodontics, College of Dentistry, Ab Shetty Institute of Dental Sciences- (Nitte to Be Deemed University), Manguluru, India; g Department of Conservative Dentistry & Endodontics, Sri Rajiv Gandhi College of Dental Sciences and Hospital, Benguluru, India

**Keywords:** Oral candidiasis, white fungus, covid-19, sars-coV-2, Mucormycosis, black fungus

## Abstract

**Introduction:**

Bacterial and fungal secondary infections following COVID-19 disease are widely being reported and are an area that should receive careful attention. Mucormycosis is a fatal fungal condition affecting immunocompromised patients caused by a group of mold mucoromycetes. *Candida albicans* (*C. albicans*) is an oral commensal present in almost 40–65% of healthy oral cavities in adults. Several cases of mucormycosis and oral candidiasis have been reported lately in COVID-19 patients, and it may elevate the associated risks of morbidity and mortality.

**Materials and Methods:**

Articles were taken from a period of 2020 to April 2021 using search sources such as Cochrane, PubMed, Fungiscope and Mycobank using keywords mucormycosis, Black fungus, oral candidiasis, white fungus, COVID-19, Sars-Cov-2.

**Discussion:**

The development of oral mucocutaneous lesions, such as mucormycosis and candidiasis in COVID-19 patients could be due to inhaling spores resulting in pulmonary and/or sinus congestion and prolonged mechanical ventilation in the ICU settings and the long-term use of broad-spectrum antibiotics respectively. The onset of candidiasis after the emergence of COVID-19 clinical signs and symptoms varied considerably and is reported within 1–30 days in most of the cases reported in the literature. Biofilms present on the denture surfaces are predisposing factors to oral candidiasis. We aim to summarize the limited data available regarding diagnosis, clinical presentation, and therapeutic approaches for the management of Mucormycosis and oral candidiasis in COVID-19 patients.

**Conclusion:**

Careful monitoring of oral lesions should be instituted through interdisciplinary telemedicine and teleconsultation to aid in primary diagnosis, thereby avoiding personal attendance during the pandemic. Dental practitioners should be included among the interdisciplinary teams for exhaustive intraoral examination and reduce the risk of morbidity and mortality.

The COVID-19 associated oral mucocutaneous lesions require an inter-disciplinary approach for their management and extensive research to identify their epidemiological importance and pathophysiology. Bacterial and fungal secondary infections following COVID-19 disease are widely being reported and are an area that should receive careful attention. Patients with COVID-19 severe illness treated in the intensive care unit (ICU) are ten times more prone to develop bacterial/fungal secondary infections than secondary viral infections.

*Candida albicans* (*C. albicans*) is an oral commensal present in almost 40–65% of healthy oral cavities in adults. Candidal infection in the oral cavity often involves an immunocompromised host [1]. Several cases of oral candidiasis have been reported lately in COVID-19 patients, and it may elevate the associated risks of morbidity and mortality. Therefore, early identification of oral candidiasis in these patients is necessary for successful and effective management.

Zygomycosis was the earlier mycotic name for the newly termed mucormycosis, a fatal fungal infection caused by mucoromycetes, a group of molds targeting immunocompromised patients, particularly those suffering from diabetes mellitus, stem cell and organ transplantation and haematological malignancies [2]. Laboratory investigations and management for this condition have been a challenge in COVID-19 patients.

The information reported in this paper was collected from published literature in PubMed, Cochrane, Fungiscope, and Mycobank database with a search performed using the keywords (COVID-19 or SARS-CoV-2), oral candidiasis, mucormycosis, black fungus and white fungus. As the presentation of oral candidiasis and mucormycosis among COVID-19 patients were observed among specific reported literature with contrasting findings, we aim to summarise the limited data available regarding diagnosis, clinical presentation, and therapeutic approaches for the management of oral candidiasis and mucormycosis in COVID-19 patients.

ROCM-Rhino-orbito-cerebral mucormycosis is the most common mucormycosis prevalent and is most commonly associated with diabetic patients and with patients suffering from diabetic ketoacidosis. It is believed that individuals inhaling spores from the atmosphere, develop sinus and/or pulmonary congestion. Furthermore, thrombo-inflammation, cytokine storm and microvascular coagulation will lead to dysbiosis in the immune response strengthening the presence of fungal infections [3]. Angioinvasion and eventual thrombosis are clinical hallmarks of mucormycosis. Susceptible individuals display periorbital facial pain, oedema of eyelids, proptosis, bilateral maxillary sinusitis, headache, tooth pain in the maxillary anterior region, and in severe cases acute vision loss. Clinicians should check for nasal discharge, development of black lesions in the palatal region of the maxilla extending towards the soft palate [3]. Radiographic imaging with CT has revealed a characteristic ‘reverse halo sign’ in patients with mucormycosis. It has been a challenge to distinguish mucormycosis from other infections. Biopsy specimens from the affected sites are needed to confirm the diagnosis. There are technical difficulties also associated with biopsies; surgeons need to be careful when taking biopsy as mucoromycetes are fragile and the culture can reveal a negative result [4]. Molecular identification using DNA probes targeting the 18S subunit, 18S-targeted semi-nested PCR, and real-time PCR targeting cytochrome b gene have aided in confirming the diagnosis for mucormycosis (Table 1) [4].

**Table 1.** Predisposing host factors associated with oral candidiasis and mucormycosis

Oral candidiasis	Local factors
	Use of dentures
	Inhaler use
	Decreased salivary flow
	**Systemic factors**
	Uncontrolled diabetes
	Immunosuppression
	Inadvertent use of broad-spectrum antibiotics/or corticosteroids
	Nutritional deficiencies*
Mucormycosis	**Local factors**
	Inhaler user
	Acute sinusitis
	**Systemic factors**
	Uncontrolled diabetes
	Immunosuppression
	Hematological malignancy
	Hematopoietic stem cell transplantation
	Solid organ transplantation**

*^5^,**^2^.

Although anti-fungal therapy has been instituted for mucormycosis, the presence of this condition in COVID-19 patients and its management has been a challenge. Nevertheless, the first line of anti-fungal therapy involves using liposomal amphotericin B and amphotericin B lipid complexes. The second line recommended is posaconazole along with a combination therapy of caspofungin and liposomal amphotericin B/amphotericin B lipid complex (Table 2). The treatment should be carried out for 4–6 weeks and maintenance therapy instituted for long-term immunocompromised cases [3].

**Table 2.** Investigations and management performed for mucormycosis and oral candidiasis

Mucormycosis	Investigation
	Radiographic Imaging using CT
	Biopsy
	DNA probes targeting the 18S subunit
	Real time PCR targeting the cytochrome b gene*
	**Management**
	First line: Anti-fungal therapy using liposomal amphotericin B, amphotericin B lipid complex.
	Second line: Posaconazole along with combination therapy of caspofungin and liposomal amphotericin B/ amphotericin B lipid complex**
	Radical resection may include partial to total maxillectomy*
**Oralcandidiasis**	**Investigation**
	Exfoliative cytology, potassium peroxide staining, imprint specimen for microbiology culture, culture analysis, salivary assays and oral mucosal biopsy***
	**Management**
	200 mg fluconazole tablets are given initially followed by 100–200 mg daily for 1–2 weeks.****
	Nystatin oral suspension 100,000 IU/ml as oral rinse and can be discontinued after 48 hours. ****

*^4^, **^3^, ***^11^, ****^15^

Dentists should be vigilant and a detailed medical history along with a thorough intra-oral examination are needed to circumvent the condition. Symptoms such as facial/sinus pain, black pigmentation and nasal discharge are signs that need to be flagged. In most reported cases, radical resection may be required, which can include partial or total maxillectomy depending on the severity and spread. Antifungal therapy along with surgical debridement are keys to control and possibly eliminate the condition [4].

Taking into consideration the characteristic disease progression and course of severe COVID-19 disease, most of the patients have associated risk factors such as broad-spectrum antibiotics and corticosteroids usage, lymphocytopenia, admission to the ICU, mechanical ventilator support, or other local risk factors such as improper oral hygiene habits, denture-wearing or decreased flow of saliva resulting in dryness of the mouth due to the use of certain medications that may favour *Candida* proliferation and infection [5]. Table 1 summarises the various predisposing host factors that are associated with the development of oral candidiasis and mucormycosis.

In case of ineffective treatment, oral candidiasis may spread from the oropharynx to the oesophagus and upper gastrointestinal tract or systemically through the bloodstream. The candidemia that occurs, as a result, has been reported to have significant morbidity and a mortality rate of 71–79% [6]. A study reported from Iran in which 53 COVID-19 patients with oropharyngeal candidiasis (OPC) were closely monitored showed cardiovascular diseases and diabetes as the predominant underlying conditions [7]. The most common risk factor associated was lymphopenia (71%). Out of the 65 *Candida* isolates causing OPC that were recovered, *C. albicans* (70.7%) was most common, followed by *C. glabrata* (10.7%), *C. dubliniensis* (9.2%), *C. parapsilosis sensu stricto* (4.6%), *C. tropicalis* (3%) and *C. krusei* (1.5%.) [7]. Patients with severe COVID-19 disease may require the support of mechanical ventilation, and a study has reported that almost 8.3% of such patients required invasive ventilation [8]. Mechanical ventilatory apparatus is associated with an increased risk of ventilator-associated pneumonia, a type of nosocomial pneumonia. This type of pneumonia is associated with the microbiota of the oropharynx and the oral cavity and has shown to have a strong association with periodontitis [9].

*Candida* can adhere to host cell surfaces which is crucial for its successful commensalism and persistence in the body during active infections. Biofilms present on the denture surfaces are predisposing factors to oral candidiasis, as *Candida* adheres to the polymethylacrylate of dentures and cracks and micro-fissures on the material favours its retention [10]. Poor oral hygiene and failure to remove dentures at night before sleep are factors that lead to biofilm formation on surfaces of dentures.

*Candida* releases certain extracellular enzymes that have a locally destructive effect on host tissues.^7^ The Secreted Aspartyl Proteinases (SAPs) produced by *C. albicans* can directly cause host cell damage, induce growth of hyphae for tissue invasion, increase adherence following receptor site exposure, and it also causes destruction of host immunoglobulins and other defense proteins [6,10]. Hemolysins produced by *Candida* are substances that break down red blood cells, and are an important attribute that helps in its survival within the host through its increased ability for iron sequestration. Hemolysin production by candida increases with increased glucose concentration, and this could be a predisposing factor for candidiasis in uncontrolled diabetics [8]. Under normal health conditions, the multiplication, and growth of candida are prevented by other organisms present in the oral microbiota, and therefore, at lower levels, it cannot bring about pathological alterations in the oral mucosa [11]. Reduced production of antimicrobial proteins such as lysozyme and lactoferrin in patients with hyposalivation can also result in decreased antifungal properties, leading to the development of oral candidiasis. The downregulation of the immune system in severe acute respiratory syndrome coronavirus 2 (SARS-CoV-2) infection, therefore, could be a causal pathway for the increased oral manifestations associated with COVID-19, mainly the ones of fungal origin [11].

The clinical presentation of pseudomembranous oral candidiasis is diagnostic because of the classic white appearance of the lesion [11]. Another relevant diagnostic feature of pseudomembranous candidiasis is that these white lesions can be wiped off by gentle scraping with gauze, leaving an underlying erythematous surface [12]. Other diagnostic methods include exfoliative cytology, potassium peroxide staining, imprint specimen for microbiology culture, culture analysis of oral swabs specimen, salivary assays, and oral mucosal biopsy. Periodic acid–Schiff staining is helpful to obtain definitive diagnosis [11]. In most of the reported cases of COVID-19, a thorough intra-oral examination and correlation with other underlying factors aids the clinician in arriving at the diagnosis of oral candidiasis.

The smear images of two COVID-19 patients who had visited a dental university clinic in Mangalore, India complaining of black spots and white patches in the nasal cavity and on the tongue respectively is shown in Figures 1 and Figures 2). The report revealed Gram stained smears showing few epithelial cells, numerous pus cells, moderate number of budding yeast cells with pseudo hyphae, numerous Gram-negative rods and moderate Gram-positive cocci in pairs suggestive of candidiasis (Figure 1), and an aseptate hyaline broad branching hyphae in KOH wet mount smear for mucormycosis (Figure 2). The onset of candidiasis after the emergence of COVID-19 symptoms varied considerably and took place within 1 to 30 days in most of the cases reported [13].

**Figure 1.**
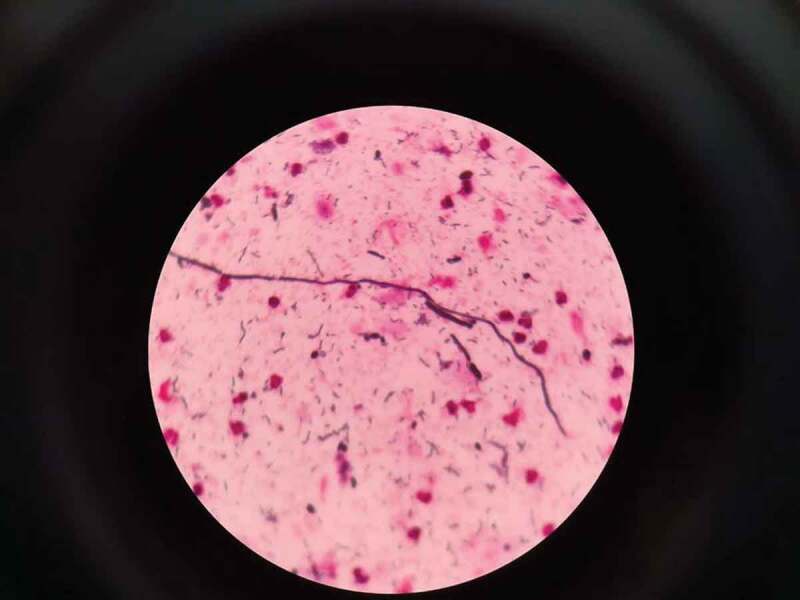
Gram stain shows budding yeast like cells with pseudo hyphae along with few epithelial cells, no pus cells, numerous Gram-negative rods and Gram-positive cocci in pairs

**Figure 2.**
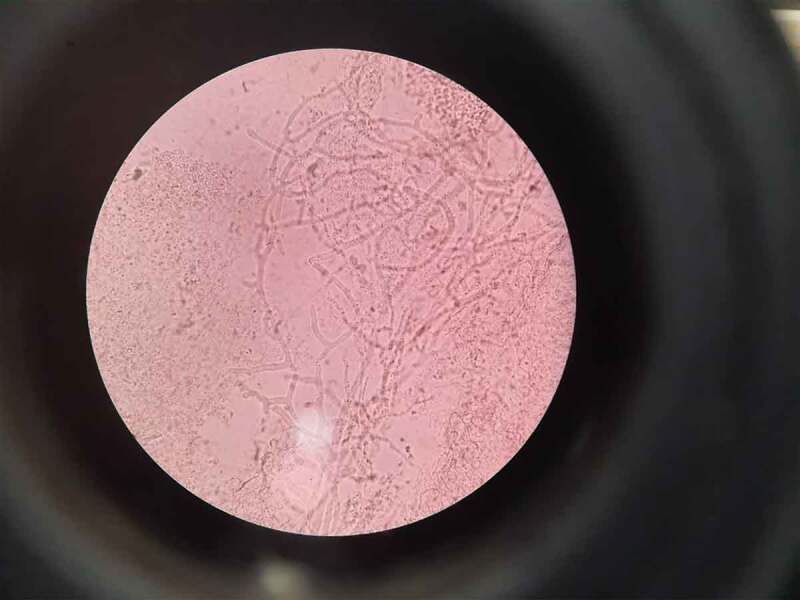
Aseptate hyaline broad branching hyphae on KOH wet mount smear for mucormycosis; site-nasal cavity

Nystatin’s oral suspension (100,000 IU/ml and 400,000–600,000 IU/ml) is available as an oral rinse for oral candidiasis treatment [11,14]. The oral suspension of nystatin can be discontinued after 48 hours following the disappearance of perioral symptoms [15]. 10 mg of Clotrimazole 10 mg tablets are also used for the management of oropharyngeal candidiasis. 200 mg Fluconazole tablets, 200 mg are used, administered on the first day of antifungal therapy, followed by 100–200 mg of fluconazole daily for the next 7–14 days [16]. Fluconazole suspension is also available as an oral rinse. Denture wearers should be advised to perform regular disinfection of acrylic partial or complete dentures by soaking them in a 0.5% sodium hypochlorite solution for 10 minutes [11,17].

The development of oral mucocutaneous lesions such as candidiasis in COVID-19 patients could be due to prolonged mechanical ventilation in the ICU settings (ICU) settings and the long-term use of broad-spectrum antibiotics and corticosteroids, which can result in immunosuppression [18]. The use of high doses and long duration of antibiotic therapy before being transferred to the ICU could have resulted in intra-oral candidiasis, ulcers and macroglossia, with oral candidiasis being most prevalent among mild to critically ill COVID-19 patients [19,20]. Furthermore, a lack of knowledge regarding treating COVID-19 and fear of a secondary infection developing results in indiscriminate prescription of antibiotics and antifungals. Moreover, high use of extended spectrum beta lactamases and incorporating an anti-fungal as an empirical therapy warrants antibiotic stewardship[20,21] Incorporating cessation of antibiotics at 48 hours of prescription, evaluating biomarkers such as galactomannan and performing culture tests will put a halt in over-zealous antibiotic prescriptions. Regarding mucormycosis, there is a lack of sufficient evidence in the literature reporting intra-oral findings, primarily mucormycosis associated with COVID-19 disease, due to the containment situation owing to the pandemic and lack of access to sufficient testing facilities to confirm the diagnosis. Therefore, careful monitoring of oral lesions should be instituted through interdisciplinary telemedicine and teleconsultation to aid in primary diagnosis, thereby avoiding personal attendance during the pandemic. Also, the dental practitioners should be included among the interdisciplinary teams dealing with severely ill COVID-19 patients in ICUs for exhaustive intraoral examination and to reduce the risk of morbidity and mortality associated with these lesions.
